# Association between social conditions and oral health in school failure

**DOI:** 10.11606/S1518-8787.2019053001457

**Published:** 2019-12-02

**Authors:** Inara Pereira da Cunha, Antonio Carlos Pereira, Marcelo de Castro Meneghim, Antônio Carlos Frias, Fábio Luiz Mialhe

**Affiliations:** I Universidade Estadual de Campinas. Faculdade de Odontologia de Piracicaba. Departamento de Ciências da Saúde e Odontologia Infantil, Piracicaba, SP, Brasil; II Universidade de São Paulo. Faculdade de Odontologia. Departamento de Odontologia Social, São Paulo, SP, Brasil

**Keywords:** Adolescent, Academic Failure, Risk Factors, Oral Health, Socioeconomic Factors

## Abstract

**OBJECTIVE:**

To evaluate the association of school failure among Brazilian adolescents with social conditions and aspects of oral health through hierarchical analysis.

**METHODS:**

A state-wide survey of 5,558 adolescents from the state of São Paulo, Brazil, inquired about the sociodemographic and social capital of participants by using a structured questionnaire. Trained and calibrated professionals performed intraoral examinations and interviews in the households. Questions about the access to dentist, reason for and frequency of using dental services, toothache episodes and impact of oral conditions on daily activities (OIDP) were applied. Oral examinations evaluated caries experience, tooth losses, periodontal problems, presence of open bite, and maxillary and mandibular overjet. School failure was estimated according to the teenagers’ years of schooling. The independent variables were grouped into three blocks: sociodemographic and economic characteristics, social capital and oral health aspects. The multiple hierarchical logistic regression model was used to identify the factors associated with school failure.

**RESULTS:**

Of the total sample, information about schooling of 5,162 adolescents was obtained, of whom 29.6% presented school failure. We found that adolescents over the age of 16 years who did not declare themselves as white, female, with feelings of insecurity, unhappiness, with toothache, caries, tooth losses, affected by dentofacial and/or periodontal changes, were more likely to fail at school.

**CONCLUSION:**

Oral disorders and social factors were associated with school failure in adolescents. A successful school trajectory was a strong determinant of health, therefore actions between the educational and health sectors must be developed for adolescents, especially those with this profile.

## INTRODUCTION

Schools are important settings for the cognitive and emotional development and health of children and adolescents^1^. When students do not attend school at the correct age when they were supposed to have done so, according to the national normative standards, due to dropping out of school, grade repetition or late enrollment in school, this phenomenon is known as school failure or educational lag^2^. According to Psacharopoulos (2007)^3^, school failure “means that a school system is failing to provide services leading to learning, or that a student is failing to advance to the next grade and eventually becomes a drop out”.

School failure compromises adolescents’ emotional well-being, is associated with the search for and use of illicit substances^3^, increases their involvement with crime, and increases their chances of experiencing teenage pregnancy^4^. For society and the state, school failure represents problems in the formation of human capital, impacting the future of a country’s economy and development^5^.

According to the Organization for Economic Cooperation and Development (OECD), the statistics on school failure in Latin America are alarming^5^. By 2015, Colombia had a higher school repetition rate (43%) than that of Brazil (36%), Uruguay (36%) and Chile (24.8%), all of which were much higher than the world average (12%) and rates in developed countries such as Finland (3%) and the United Kingdom (2.8%)^5^.

In Brazil, there are over 35 million students enrolled in basic education. High school is the stage of basic education where there is the highest percentage of students with two or more years of school delay. There are more than 2.2 million adolescents in a state of school delay, who at some point were either not approved for proceeding to the next grade, or evaded school, returned later and were included into a certain grade not matching their age. School delay is part of the school failure process, which affects mainly the most vulnerable sections of the population^6^. A successful school trajectory is a strong determinant of health and life expectancy^7^, however, in order to guarantee this right, it is necessary to know the factors associated with school failure.

The causes of school failure have attracted attention from different areas of knowledge, from social scientists to health researchers ^2 , 3 , 8 - 11^. It is known, for example, that the risk of dropout in high school is higher among low-income adolescents^8^, and it is also influenced by gender^9^, race/ethnicity^10^and the social context in which students are inserted^11^. In addition, studies have indicated that the poor health status of students may impair their cognitive development and participation in school activities, increasing the rates of school failure^12^.

In the dental context, there is some evidence that dental caries, toothache, gingivitis and dentofacial features are oral disorders that compromise academic activities, increasing absenteeism and decreasing the school performance of children and adolescents^13 - 15^. However, nothing is known about the impact of oral changes on school failure.

Therefore, the aim of this study was to evaluate the association of sociodemographic factors, social capital aspects, and oral health status with adolescents’ school failure.

## METHODS

This was a cross-sectional study conducted in the state of São Paulo, one of the largest Brazilian states in terms of population, with 45.1 million inhabitants comprising 21.7% of the country’s population. In 2015, a state-based oral survey known as SB São Paulo 2015 was conducted encompassing all regions of the State^16^. Initially, 177 municipalities were selected, plus the capital (the city of São Paulo). At a second stage, 390 Census Sectors were selected (2 sectors for 177 municipalities and 36 sectors for the city of São Paulo). The sample plan was prepared by a conglomerate method for a two-stage lottery with probability proportional to the size of the population. A total of 17,560 people were examined in 163 municipalities for the different segments evaluated. Initially, 7,126 adolescents from 15 to 19 years of age were selected (by lottery). However, 22% (1,568 of the adolescents) refused to participate in the survey, so a final sample of 5,558 adolescents remained. This study was approved by the FOP UNICAMP Ethics and Research Committee with Human Beings, number 1,211,025 (2015), CAEE No. 46788215.9.0000.5418, according to Resolution 466/12 of the National Health Council, concerning research on human beings.

A total of 250 work teams organized by the municipalities participated in the study. Each team was composed of a dentist and a dental assistant. The training and calibration process of the teams followed the guidelines proposed by the WHO^17^by calculating the concordance between each examiner and the results obtained by the team consensus. The examiners who obtained an intra-examiner and inter-examiner agreement Kappa statistic with a minimum value of 0.65 participated in this study.

At a first time interval, a team of surveyors in each municipality visited all households in the census tracts, which corresponded to territorial spaces divided by the Brazilian Institute of Geography and Statistics (IBGE). Thus, the residents eligible for the SB São Paulo 2015 research (individuals of 15–19, 35–44 and 65 years of age) were identified. The individuals were informed of the research, and those who presented the necessary characteristics were noted on the survey record sheet.

At a second time interval, the teams of examiners (dentists and assistants) went through the census sectors and the households visited by the surveyors; they invited the individuals to participate in the research, asked them to sign the Term of Free and Informed Consent, performed the exams and held the interviews. These actions continued until the predicted number of participants for each sector was fulfilled. If this did not occur, they went through the sector again. All information items about the residents examined/interviewed, including those who were absent and those who refused to participate in the study, served to calculate the rate of densification per census sector and the rate of non-response. More information about the data collection can be found in the final report of the SB São Paulo survey 2015^16^.

Initially, the dentists applied a questionnaire to the adolescents; this contained questions enabling the demographic and socioeconomic characterization of the participants (age, sex, skin color, family income and family agglomeration). In the *SB de São Paulo* survey, the demographic data included information about the sex, age and race/skin color, according to the classification scheme proposed by the Brazilian Institute of Geography and Statistics, into white, brown, black, yellow and indigenous. Thus, we categorized skin color into white and non-white (black, brown, yellow and indigenous). Family income was categorized according to median sample (≤ US$ 375.00 and > US$ 375.00) and family agglomeration (≤ 1.5 to > 1.5 persons per room).

Social capital was investigated based on three questions suggested by Grootaert et al. (2003)^18^. The first was as follows: “If there were a water supply problem in this community, how likely would it be that people would cooperate to try to solve the problem?” The answers to this question were dichotomized as likely and unlikely. Another question was as follows: “In general, how do you feel about crime and violence when you are alone at home?” The answers to this question were dichotomized as safe and unsafe. Finally, the participants were asked about their current level of happiness. The answers were categorized as happy and unhappy.

The access to dental services and the reason for access were evaluated by the following questions: “Have you ever been to the dentist’s office?” and “What was the reason for your last visit?” The first question had yes and no answers, and the second was dichotomized into review/prevention/check-up and dental/other treatment. Toothache was diagnosed by means of the question “In the last six months, did you have toothache?” The answers were categorized into yes, no and non-respondents.

In addition, we implemented the instrument Oral Impacts on Daily Performance (OIDP), which investigates the impact of nine items related to daily activities that may be affected by oral conditions^19^. The answer options were no, yes, do not know, or did not want to respond, which was treated as missing information. The OIDP was analyzed as a dichotomous variable, considering the impact of the presence of at least one of the nine questions marked as positive.

Subsequently, oral exams of the adolescents were performed under natural light, using a periodontal probe (CPI), flat mouth mirror and wooden spatulas according to WHO recommendations^17^. Decayed teeth and tooth loss were evaluated by the carious and lost components of the carious, lost and restored teeth index (Index DMFT - decayed, missing and filled teeth). The presence of caries and tooth loss were categorized as either none or at least one decayed or lost tooth. Periodontal changes were evaluated by the Community Periodontal Index (CPI) considering the index teeth (16, 11, 26, 36, 31, 46) for the 15–19 age group^17^.

The following dentofacial changes were examined: maxillary overjet less than 1 mm (decreased) or greater than 3 mm (increased), mandibular overjet and anterior open bite. Maxillary overjet was measured according to the vestibular face of the lower incisors to the upper incisor. The mandibular overjet was measured in the opposite direction. The anterior open bite was measured by the distance between the incisal edges of the upper and lower incisors and was considered present when the measured distance was greater than zero mm^20^. The WHO CPI probe was used to perform measurements.

School failure was the outcome variable of the study and was evaluated by the following question: “Up to which grade did you study?” The answers were converted into years of study considering the first year of elementary school and repetitions^2 , 3 , 6^. To differentiate adolescents with at least two years of delay with regard to progress expected by age, the criterion of 11 years of studies was adopted for adolescents 18 to 19 years; 10 for those aged 17; 9 for those aged 16; and 8 for those aged 15, as performed in the study by Barbato et al. (2009)^21^.

**Figure 1:**
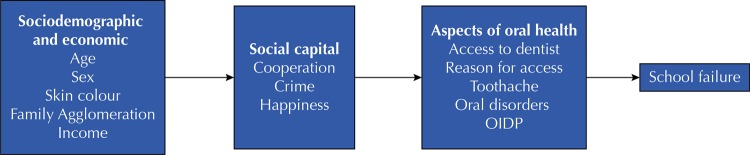
Hierarchical conceptual model for the association between social factors, aspects of oral health, and school failure. Note: For the purpose of analysis, the variables were divided into blocks: (a) block 1, (b) block 2, and (c) block 3, adapted from Kaewkamnerdpong and Krisdapong (2018)^15^.

The analyses were performed using the “SURVEYFREQ” and “SURVEYLOGISTIC” procedures of the SAS program, considering the complex sampling plan. We initially calculated the weighted prevalence of school failure with the respective 95% confidence interval, considering the total sample. The sample was dichotomized as schoolchildren with and without delay, and analyzing the association of this outcome variable with the other variables were performed using weighted hierarchical multiple and simple logistic regression models. A hierarchical model was developed to demonstrate the relationship between variables ( Figure 1 ). Under this hierarchization, block 1 was formed by sociodemographic conditions, among them, age, sex, skin color, income and family agglomeration. Block 2 contained questions related to social capital. The block 3 was constructed with information referring to aspects of oral health, such as access and reason for dental consultation, toothache, presence of decayed teeth, dental loss, dentofacial and/or periodontal changes, and impact of oral conditions in daily activities. The variables with p ≤ 0.20 in each block were tested with the multiple logistic regression model, and model entry occurred from the first to the third block, with such variables remaining in the model that continued to be associated with the delay with p ≤ 0.05 after adjustment to the variables of the same block and to the hierarchically superior variables. From the weighted logistic regression analyses, the crude and adjusted odds ratios were estimated with the respective 95% confidence intervals.

## RESULTS

Of the 5,558 adolescents, 92.6% participated in the survey of information about school failure (n = 5,162). In the sample, the percentage of students with school failure was 29.6% (n = 1,528). The weighted prevalence of students with school failure was 30.1% (95% CI: 29.4%–30.8%).

Table 1 shows the weighted individual analyses of the associations between school failure and the analyzed variables by weighted prevalence (standard error). It was observed that most of variables presented p < 0.20 in the bivariate analysis of the association with school failure ( Table 1 ).

**Table 1:** Weighted individual analyses of the association between school failure and the analyzed variables by weighted prevalence (standard error)

Variable	Category	n(%)^#^	School Failure	^*^OR gross (^$^IC95%)	p-value
			Yes^& ^	^$^($IC95%)	
			Weighted prevalence (standard error)		
Block 1					
Age	Up to 16 years	2591 (50.2%)	13.8% (0.22%)	Ref	
	More than 16 years	2571 (49.8%)	45.4% (0.32%)	5.18 (4.78-5.62)	< 0.001
Sex	Male	2255 (43.7%)	25.2% (0.22%)	Ref	
	Female	2907 (56.3%)	33.9% (0.38%)	1.52 (1.40-1.65)	< 0.001
Skin colour	White	3067 (59.4%)	28.3% (0.37%)	Ref	
	Non-whites ª	2095 (40.6%)	32.8% (0.29%)	1.23 (1.14-1.33)	< 0.001
Income	Up to US$ 375	1791 (43.1%)	30.7% (0.33%)	1.00 (0.94-1.06)	0.912
	More than US$ 375	2365 (56.9%)	30.6% (0.36%)	Ref	
Agglomeration	Up to 1.5	1790 (35.0%)	29.2% (0.34%)	Ref	
	more than 1.5	3331 (65.0%)	30.6% (0.42%)	1.07 (0.98-1.18)	0.150
Block 2					
Cooperation	Likely	3526 (68.6%)	28.8% (0.42%)	Ref	
	Unlikely	1615 (61.4%)	32.6% (0.31%)	1.19 (1.11-1.27)	< 0.001
Crime	Safe	3434 (66.8%)	27.3% (0.28%)	Ref	
	Unsafe	1707 (33.2%)	35.1% (0.28%)	1.44 (1.37-1.52)	< 0.001
Happiness	Happy	4766 (92.7%)	29.6% (0.30%)	Ref	
	Unhappy	375 (7.3%)	35.5% (0.15%)	1.31 (1.17-1.47)	< 0.001
Block 3					
Access to dentist	No	178 (3.5%)	30.4% (0.13%)	1.02 (0.72-1.42)	0.931
	Yes	4938 (96.5%)	30.1% (0.31%)	Ref	
Reason for access	Review	1911 (39.2%)	28.5% (0.26%)	Ref	
	Treatment	2965 (60.8%)	31.1% (0.30%)	1.13 (1.06-1.21)	0.002
Toothache	No	3817 (74.6%)	28.9% (0.33%)	Ref	
	Yes	1297 (25.4%)	33.7% (0.27%)	1.25 (1.17-1.34)	< 0.001
Decayed teeth	No	30.28 (58.7%)	29.6% (0.29%)	Ref	
	Yes	2134 (41.3%)	30.8% (0.27%)	1.06 (1.01-1.11)	0.010
Tooth loss	No	4489 (90.0%)	29.2% (0.38%)	Ref	
	Yes	673 (13.0%)	36.4% (0.17%)	1.39 (1.22-1.58)	< 0.001
Dentofacial features	No	2436 (47.2%)	26.8% (0.34%)	Ref	
	Yes	2726 (52.8%)	33.0% (0.32%)	1.35 (1.28-1.42)	< 0.001
Periodontal changes	No	3054 (59.2%)	29.7% (0.23%)	Ref	
	Yes	2108 (40.8%)	30.6% (0.31%)	1.04 (0.99-1.10)	0.096
OIDP	No impact	3274 (63.9%)	28.7% (0.26%)	Ref	
	With impact	1848 (36.1%)	32.6% (0.28%)	1.21 (1.14-1.27)	< 0.001

^#^In the sample; * Odds ratio; ^$^ 95% confidence interval; ^&^ Reference level; ª The category non-whites included black (18.1%), yellow (2.6%), brown (78,7%) and indigenous (0.6%) participants.

In the adjusted model ( Table 2 ), it was observed that age, sex and skin colour of the adolescents were demographic characteristics that remained associated with school failure. With regard to social capital, adolescents who did not feel safe and did not feel happy remained associated with school failure. In relation to clinical characteristics, those adolescents with toothache, decayed teeth, tooth loss, dentofacial problems and periodontal problems were also more likely to fail in school.

**Table 2:** Weighted multiple analysis of the association between school failure and the analyzed variables.

Variable	Category	Model 1		Model 2		Model 3	
		^*^OR adjusted (^$^IC95%)	p-value	^*^OR adjusted (^$^IC95%)	p-value	^*^OR adjusted (^$^IC95%)	p-value
Block 1							
Age	Up to 16 years	Ref		Ref		Ref	
	More than 16 years	5.12 (4.72-5.55)	< 0.001	5.07 (4.68-5.50)	< 0.001	5.42 (5.00-5.87)	< 0.001
Sex	Male	Ref		Ref		Ref	
	Female	1.44 (1.31-1.58)	< 0.001	1.42 (1.30-1.55)	< 0.001	1.34 (1.23-1.47)	< 0.001
Skin colour	White	Ref		Ref		Ref	
	Non-whites	1.29 (1.01-1.24)	0.037	1.30 (1.19-1.43)	< 0.001	1.32 (1.20-1.44)	< 0.001
Block 2							
Crime	Safe			Ref		Ref	
	Unsafe			1.25 (1.17-1.34)	< 0.001	1.27 (1.19–1.35)	< 0.001
Happiness	Happy			Ref		Ref	
	Unhappy			1.25 (1.15-1.36)	< 0.001	1.37 (1.26–1.49)	< 0.001
Block 3							
Toothache	No					Ref	
	Yes					1.09 (1.03-1.16)	0.017
Decayed teeth	No					Ref	
	Yes					1.15 (1.08-1.23)	< 0.001
Tooth loss	No					Ref	
	Yes					1.14 (1.03-1.27)	0.010
Dentofacial features	No					Ref	
	Yes					1.40 (1.31-1.50)	< 0.001
Periodontal changes	No					Ref	
	Yes					1.32 (1.23-1.40)	< 0.001
Model Fit Statistics	AIC (null model =23441464)	20894932		20742232		20431512	
	-2 log L (null model =23441462)	20894922		20742220		20431490	

*Odds ratio; ^$^Confidence Interval 95%.

## DISCUSSION

This study demonstrated that demographic, social and oral variables were associated with school failure in adolescents. To the best of our knowledge, this is the first study to investigate this association, a fact that brings new evidence to the importance of oral health professionals in the school context.

Among the sociodemographic variables investigated, the age of adolescents was a condition associated with school failure. Adolescents older than 16 years were five times more likely to fail in school. A recent national survey conducted in Brazil also showed school inequalities according to the age of adolescents^22^. It was observed that 68.4% of the youngest people between 15 and 17 years old were attending school at the correct age and there were only 31.7% of people aged 18 to 24 years in this situation in 2017^22^. These findings indicated that there were important disparities in school failure in adolescents associated with demographic and social demands as they grow older, such as having to work, having to take care of household chores or even losing interest in studying ^2 , 3 , 22^.

Sex was another variable associated with school failure. A population-based study performed in Brazil demonstrate that in 2015, of the total of 1.3 million young people aged 15 to 17 who did not go to school, 610,000 were female. Among them, 35%, or 212,000 in this age group were already mothers and only 2% of adolescents who became pregnant continued to be enrolled in schools^23^. This is relevant information, since adolescents who experience pregnancy or maternity can suffer from diverse health and time limitations that discourage them from continuing with their studies. In the first months after childbirth, the school setting has to be adapted, e.g. by developing a differentiated schedule or creating strategies that make it easier for the young mothers to take care of their babies^4^. A study conducted in Colombia, for example, revealed similar characteristics among young people who had dropped out of school, the majority of whom were teenagers, and women with at least one child^8^.

Evidences from Brazil^22 , 23^ and international assessments^9^ have demonstrated that there were inequalities among the white, black and mixed populations with regard to school failure, which was also observed in the present study. According to Bécares et al. (2015)^10^, the unsatisfactory school performance among adolescents of racial minorities goes beyond economic differences, in that school performance in these groups is mainly threatened by negative stereotypes. The authors strongly recommended combating racial discrimination as a tool for improving the success of equality in school.

It is important to emphasize that the well-being of adolescents also involves the social context in which they find themselves. Research has shown that adolescents exposed to community violence become vulnerable and are more likely to acquire depressive and post-traumatic symptoms that compromise their school performance^10 , 24^. Community violence involves the individual’s social or environmental context and may be related to aggression, assault, rape, and robbery, with or without the presence of weapons, in the neighborhood or area near the individuals’ homes^25^. As has been reported in previous research^26^, it is understandable that the feeling of insecurity identified among adolescents contributed to their school failure, as observed in the present study. In addition, the association between school failure and the feeling of happiness observed in this study are also plausible because high levels of happiness among teenagers stimulate a healthier life and better school performance^27^. This evidence indicates that happiness drives motivation and commitment to studies, playing an important role in helping students adapt to adverse learning situations^27^.

School failure has previously been shown to be associated with the general health of students^7^. However, to date, this association has not been investigated in the dental field. This hypothesis, confirmed in this study, emerged as a variable of interest due to the previously known relationship between oral problems and poor school performance^12^. In general, children with oral problems are more likely to miss classes and not perform the tasks required in school^12 - 14^. Caries and toothache, specifically, cause inability to concentrate and are associated with feelings of embarrassment and anxiety that can affect daily behavior^28^. Among adolescents, the effects of oral problems are associated with shyness and feelings of unhappiness and uselessness, which compromise their psychosocial well-being. All of these results can impact in the dropout rates of school and performance in studies concerning adolescents^11^.

We also observed an association between periodontal changes and school failure. It seems that periodontal changes impact the adolescent’s quality of life and compromise the appearance of the teeth and mouth^29^. A similar explanation elucidated the association of dentofacial changes and tooth loss with outcome. Among the oral changes investigated in this study, dentofacial features were more likely to contribute to school failure. Dentofacial changes are conditions that mainly compromise facial aesthetics, which can lead to bullying by peers in the school environment, and consequently discourage participation in school activities^30^. A study investigating the association between dentofacial changes and bullying among 920 Jordanian schoolchildren demonstrated that 47% of them were bullied due to the dentofacial features evaluated in the present study, including anterior open bite (14.1%), prominent mandibular anterior teeth (4.2%), retrognathic mandible (3.2%), and prognathic mandible (2.5%). Many schoolchildren reported that they missed classes and did not like going to school because they felt intimidated by peers^30^This finding once again reinforces the tendency of young people to feel the impact of oral conditions in a social-emotional way, which may impact their overall quality of life.

Due to the fact that this study had a cross-sectional design, it was not possible to define the causal relationship between the analyzed variables, and therefore, cohort studies are necessary to establish a causal relationship. In addition, it is known that school failure is a consequence of multiple factors such as disciplinary problems, family structure and school conditions that were not investigated, limiting the findings of this study. Despite these limitations, the large representative sample and the robust statistical analysis used in this investigation allow us to present important new evidence about the association between school failure and demographic, social and oral health variables.

## CONCLUSION

School failure was associated with demographic characteristics, social conditions and oral disorders in adolescents.
